# Transmission frequency of COVID-19 through pre-symptomatic and asymptomatic patients in AJK: a report of 201 cases

**DOI:** 10.1186/s12985-021-01609-w

**Published:** 2021-07-03

**Authors:** Majid Mahmood, Noor-ul-ain Ilyas, Muhammad Faraz Khan, Muhammad Naseem Hasrat, Nicholas Richwagen

**Affiliations:** 1 Department of Zoology, University of Poonch Rawalakot, Rawalakot, AJK Pakistan; 2 Department of Botany, University of Poonch Rawalakot, Rawalakot, AJK Pakistan; 3 Sheikh Khalifa Bin Zayd Al-Nahyan DHQ Hospital, Rawalakot, AJK Pakistan; 4 College of Medicine, University of Central Florida, Florida, USA

**Keywords:** COVID-19, COVID-19 transmission, COVID-19 patients, Pre-symptomatic, Asymptomatic, AJK, Pakistan

## Abstract

**Background:**

The COVID-19 pandemic is a catastrophic global phenomenon, affecting human life in a way unseen since the 1918 influenza pandemic. Effective management of this threat requires halting transmission, a strategy requiring accurate knowledge of SARS-CoV-2 transmission patterns.

**Methods:**

This was a retrospective contact study aiming to estimate the transmission rate of COVID-19 by tracing contacts in symptomatic, pre-symptomatic, and asymptomatic patients. History of patients’ contacts during 24 h before appearance of symptoms or infection confirmation was traced for disease transmission.

**Results:**

Overall, a total of 201 COVID-19 patients had contact with 7168 people in 24 h with an average of 35.66 contacts per patient, ranging from a minimum of 4 to maximum of 87 contacts (meetings). Out of 7168 persons met, infection was detected in 64 (0.89%). For the 155 symptomatic patients, a total of 5611 contacted persons were traced before appearance of symptoms (pre-symptomatic) in last 24 h with an average of 36.20 meetings per patient. The infection was transmitted in 63 (1.12%) people with 5548 (98.88%) remaining uninfected. Out of the 63 transmissions, 62 (98.4%) were traced within 6 h before symptom onset, while only 1 was identified in the 6–12 h timeframe before symptoms. A total of 1557 persons were traced having meeting/contacts with asymptomatic cases in last 24 h before infection confirmation. Out of these 1557 persons, only 1 was found to be infected and the infection rate was calculated to be 0.06%. Statistically, the transmission rate by pre-symptomatic patients was found to be significantly higher than the transmission rate by asymptomatic individuals (*P* < 0.05).

**Conclusion:**

In the studied population, the risk of pre-symptomatic and asymptomatic transmission of COVID-19 was low, with transmission risks of 1.12% and 0.06% respectively. Pre-symptomatic infection becomes very rare in contacts made longer than 6 h before onset of symptoms. The infection transmission is traced as long as about 9 h before the appearance of clear symptoms in the patients, but the incidence rate was as low as about 0.02% of the total contacts in that period.

## Background

The coronavirus disease (COVID-19) is caused by the severe acute respiratory syndrome coronavirus 2 (SARS-CoV-2). This illness has been declared as “once-in-a-century” pandemic and has affected practically the entire human population [1]. To date (December 28, 2020), more than 79.67 million cases have been confirmed worldwide and the cumulative deaths have exceeded 1.76 million [2]. The exponential rate of transmission has proven devastating in managing and containing viral spread [3]. Contributing to this rate is the significant fraction of patients who remain without symptoms during their entire infection course-*asymptomatic patients*. Moreover, some infected persons may only develop symptoms after a period of varying length, during which they are described as *pre-symptomatic* [4–11]. Asymptomatic and pre-symptomatic patients often go undetected and frequently encounter others without sufficient protective measures.

The extent to which COVID-19 is transmissible in pre-symptomatic and asymptomatic patients has not been addressed comprehensively, although a number of reports validate the risk of asymptomatic and pre-symptomatic transmission of SARS-CoV2 [12–22]. However, most previous studies reporting asymptomatic and pre symptomatic transmission are based on single or few cases and have many limitations [22]. Moreover, the exact transmission risk of encountering pre-symptomatic or asymptomatic patients remains to be determined, and evaluating such risk can be difficult to evaluate and quantify. Studies have varied wildly in their estimates of asymptomatic carrier numbers and transmission rates. Gauging actual proportions of such individuals (and associated risks) is therefore very difficult in any population. Additionally, methodology varies between studies [23, 24].

Despite global efforts at containment, SARS-CoV-2 continues to find new hosts and continued transmission appears unstoppable. Vaccines alone will not be sufficient as a means of control considering ongoing challenges in mass production and fair distribution [25]. Recent developments of more transmissible mutants reinforce the need for greater insight into infection spread [26]. The most promising control strategy remains blocking transmission, a method which requires accurate knowledge of transmission patterns and sources of infection. Real case-to-case studies on pre-symptomatic and asymptomatic patients in different populations are required to accurately assess the true transmission rate of COVID-19. The present study was designed and conducted to assess and estimate the rate of transmission through asymptomatic and pre-symptomatic subjects from a selected region of Azad Jammu and Kashmir, Pakistan.

## Methods

### Study design and study area

The present study was combined from 201 individual case studies, retrospectively conducted by contact trace strategy. A total of 155 symptomatic and 46 asymptomatic COVID-19 patients belonging to different areas of districts Poonch and Sudhnuti, Azad Jammu and Kashmir (AJK) were included. The study was conducted in the period of September through November 2020 in which the positive patients from May 2020 to September 2020 were traced and enrolled retrospectively.

### Patients' selection and records acquisition

PCR positive Patients were identified and traced from three hospitals: (1) Sheikh Khalifa Bin Zayd Al-Nahyan Hospital, Rawalakot, (2) District Headquarter Hospital, Palandari, and (3) Tehsil Headquarter Hospital, Hajira. Patient tracing records were provided by these hospitals as well as the district health and surveillance officers of districts Poonch and District Sudhnuti, AJK. Obtained patient records included name, gender, age, home address, telephone number, profession, quarantine or admission duration, symptoms, PCR test report, and admission or quarantine outcome. After retrieving and assessing records, 386 COVID 19 PCR positive patients were initially identified as candidates for study enrolment.

### Final selection of patients and inclusion in study

All the patients who were positive for COVID-19 by real time PCR were selected initially. The patients admitted in hospital or quarantined with symptoms were enrolled after complete recovery while the patients without symptoms were included after 14 days of their PCR test. Only patients who were able to meet with the investigator were included. Of the initially selected potential candidates (386), only 201 (52%) would ultimately be enrolled and interviewed as study participants.

**Fig. 1:**
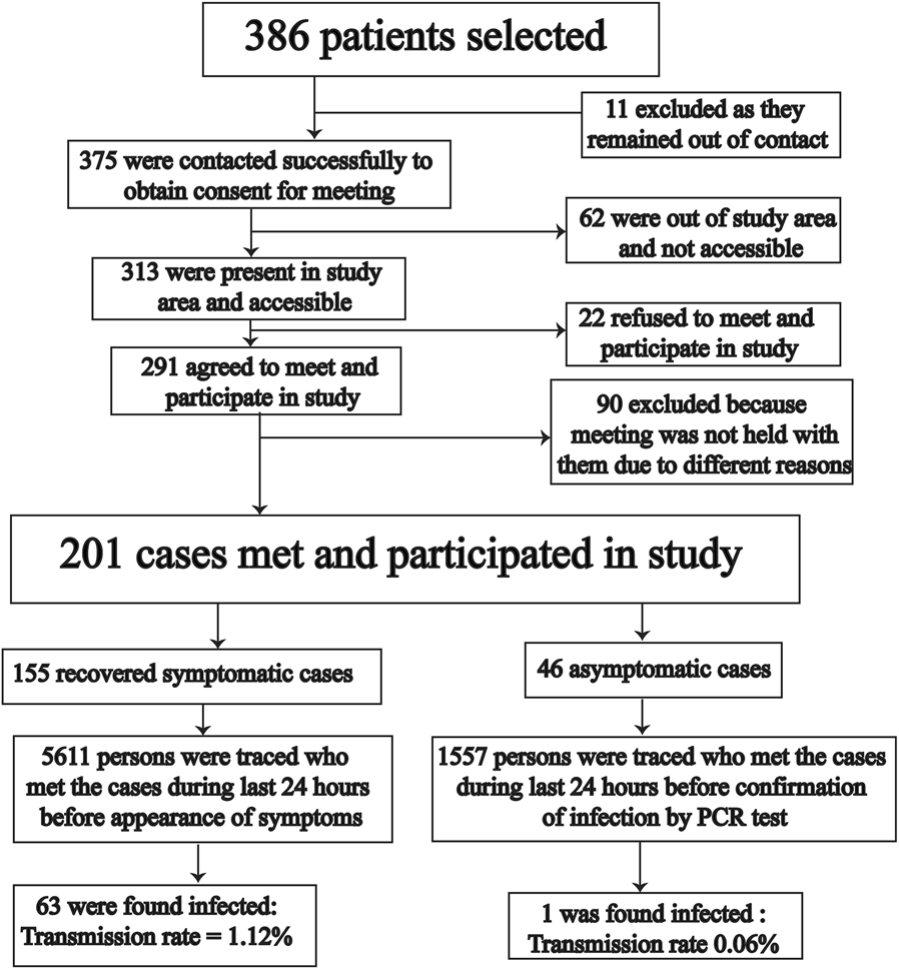
The flow chart of study methodology with inclusion and exclusion of study participants

Out of the initial 386, 375 patients were successfully contacted in order obtain consent for meeting and interview; 11 individuals could not be reached. Of the 375 contacts, 313 were locally present and accessible in the study area. Of these, 291 individuals agreed for meeting and study participation (Fig. 1).

### Meetings with patients and interviews

Successful interviews were conducted with only 201 individuals. These included 155 symptomatic and 46 asymptomatic patients. Meetings were conducted in different locales according to convenience for both patients and investigators. The meeting places included patient homes, the participating hospitals, and the Zoology Department at the University of Poonch Rawalakot (UPR).

A prepared questionnaire and data sheet were used to record information and data during interviews. Collected information included the following: *patient name, age, gender, area of residence, profession, place of job or study, date of appearance of symptoms, date of infection confirmation by PCR, possible date of infection, possible source of infection, history of hospital admission or quarantine, persons met in last 24 h before symptom appearance (in symptomatic patient cases) and before viral confirmation by test (in case of asymptomatic patients), persons meeting in different retrospective periods 0–24 h prior to symptom appearance or infection confirmation, the method of contact with meeting persons and, the detail of meeting persons to patient* (Fig. 1).

### Data of meeting persons to trace the transmission

Detailed information regarding patient person-to-person contacts was obtained during meetings. Individuals who had been contacted were then informed and traced with the help of the patient participants. Total 7168 individuals (*patient contacts*) had been contacted by the initial patient group. Data was collected on these persons. The majority of patient contacts were found to be family members, close relatives, friends, colleagues, or neighbors of the initial patients. Data collected on patient contacts included their quarantine history, PCR testing status, appearance of symptoms, and their current health status to confirm the transmission. The data of patient contacts was obtained from the original participating patient or from meetings and telephone calls on the base of availability and accessibility (Fig. 1).

### Laboratory diagnosis

Patient contacts were randomly selected for laboratory based diagnosis. Nasopharyngeal swabs were collected for this purpose and RNA was extracted by an auto-extractor (TANBead Nucleic Acid Extractor, SLA 32, Taiwan) and, then amplified and detected by real time PCR system (SaCycler-96 real time PCR system, CE IVD medical equipment, Italy).

### Follow up

All contacts were followed up for a mean period of 84.0 ± 43.0 days (23–200) to trace any symptom. In case of any symptom appearance in this duration, PCR test was performed.

### Informed consent and ethics approval

Only patients who agreed to participate were included in the study. Each patient was informed about the use of their data and all participants signed a written informed consent. The study was approved by the institutional ethics committee of University of Poonch Rawalakot (UPR/HAEC/2020/M2/C11).

### Data analysis

All the data was entered in MS Excel data sheets. Means, median, standard deviation, minimum value, maximum value, and percentages were calculated for different parameters and infection transmission rate. The transmission rate was calculated by simple percentage of infected contacts out of total contacts for total, pre-symptomatic, and asymptomatic contacts. Significance of transmission rate was calculated between pre-symptomatic and asymptomatic contacts by Pearson's Chi square test using SPSS version 16.0.

## Results

This study traced the transmission of COVID-19 by 201 cases including 126 male (62.7%) and 75 female (37.3%) patients. For each of the 201 initial patients, a separate case study was conducted which included 155 pre-symptomatic and 46 asymptomatic patients. A total of 7168 individuals (patient contacts) who met with these 201 initial patients were identified. Patient contacts had been exposed to one of the initial patients 24 h before the appearance of symptoms or confirmation of infection in the initial patient (mean meetings per patient = 35.66).

Out of 7168 contacts, 64 individuals were found to have been infected with COVID-19 with a transmission rate of 0.89% (Table 1). No case of infection was observed in the remaining 7104 contacting persons even after a mean duration of 84 ± 43 days, with minimum of 23 days from the date of infection confirmation in initial patients. Out of the total 64 infected individuals, 63 were recorded to have been infected from pre-symptomatic patients while only 1 patient was traced to acquire infection from asymptomatic patients (Fig. 1). The net rates of pre-symptomatic and asymptomatic transmissions were found to be 1.12% and 0.06% respectively (Table 1). Transmission of infection was found to be very low by pre-symptomatic patients and almost singular by asymptomatic patients. Statistically, the transmission rate was found to be significantly lower by asymptomatic individuals as compared to the transmission rate by pre-symptomatic patients (*P* < 0.05).

**Table 1:** Detail of pre-symptomatic and asymptomatic transmission of COVID-19 in contacts within 24 h prior to appearance of symptoms or confirmation of infection

	Total contacts	Average contacts	Min–Max	Infection transmitted	Infection not transmitted	Mean days from confirmation	Min–Max
Total (n = 201)	7168	35.66	4–87	64 (0.89%)	7104 (99.11%)	84.0 ± 43.0	23–200
Pre-symptomatic (n = 155)	5611	36.20	4–87	63 (1.12%)	5548 (98.88%)	85.32 ± 43.5	24–210
Asymptomatic (n = 46)	1557	33.85	6–84	1 (0.06%)	1556 (99.94%)	82.74 ± 42.5	23–180

### Pre-Symptomatic transmission

Of 201 cases, 155 patients developed symptoms. This group had 5611 pre-symptomatic contact events 24 h before the onset of symptoms. Mean persons meeting a pre-symptomatic patient was calculated to be 36.20. Out of these 5611 people contacted, 5548 (98.88%) remained uninfected even after random testing on or after day 14 and over a mean time duration post-contact of 85.32 ± 43.5 days ranging from minimum of 24 days to maximum of 210 days, while 63 of these individuals (1.12%) were tested positive. Transmission by pre-symptomatic patients was observed to be very low (1.12%) (Tables 1, 2).

**Table 2:** Detail of transmission traced by pre-symptomatic and asymptomatic COVID-19 patients in AJK

Patient type	Time duration before symptoms or confirmation	Total meetings	Average meetings	Infections transmitted	Transmission percentage
Pre-symptomatic (n = 155)	Total (0–24 h)	5611	36.20	63	1.12
	0–6 h	1544	9.96	62	4.01
	6–12 h	1410	9.09	1	0.07
	12–18 h	1285	8.29	0	0.00
	18–24 h	1372	8.85	0	0.00
	Total 6–24 h	4067	–	1	0.02
Asymptomatic (n = 46)	Total (0–24 h)	1557	33.85	1	0.006
	0–6 h	353	7.67	0	0.00
	6–12 h	436	9.48	1	0.23
	12–18 h	478	10.39	0	0.00
	18–24 h	290	6.30	0	0.00

Time line of contact event was as follows: Patients who would later develop symptoms (pre-symptomatic patients) made contact with 5611 persons. Of these persons (patient contacts), 1544 were contacted 6–0 h prior to symptom onset, 1410 were contacted 12–6 h prior, 1285 were contacted 18–12 h prior, and 1372 were contacted 24–18 h prior.

The majority of the transmission events (98.4%) occurred in meetings just before the appearance of symptoms while very rare transmission occurred in meetings before 6 h or more prior to symptom appearance (Table 2). Sixty two (62) of the 63 transmissions (98.4%) were traced in the period of 0–6 h before appearance of symptoms while 1 transmission (1.6%) was traced to a contact which occurred 6–12 h before appearance of symptoms (Table 2).

The transmission rate was calculated to be about 4% during 0–6 h before symptom appearance, while it was only 0.02% in contacts prior to 6–24 h of the symptoms (Table 2). The risk of getting infection from a pre-symptomatic patient 6 h or more from symptom onset therefore was very rare.

### Asymptomatic transmission

A total of 46 out of 201 positive cases never developed any disease symptoms during the study even after a mean duration of 82.74 ± 42.5 days after testing positive, ranging from minimum 23 days to maximum 180 days after tested positive. These cases were designated as asymptomatic. A total of 1557 persons were traced who had meetings with asymptomatic patients prior to the patients testing positive. Of these 1557 individuals, only 1 person tested positive. Interestingly, this individual had met with an infected patient for 2 days and developed mild symptoms of COVID-19 just 3 days after the first meeting. All of other traced persons (1556 out of 1557) remained asymptomatic throughout the study and were never tested positive. The transmission rate by asymptomatic patients was therefore found to be 0.06% (Table 2).

### Some selected case reports

Separate case studies were conducted for each of the 155 pre-symptomatic and 46 asymptomatic positive patients. Here we discuss 4 pre-symptomatic cases and 4 asymptomatic cases as examples selected out of total 201 cases (Table 3).

**Table 3:** Detail of 8 selected case reports out of the total 201 cases in the study

Case No	Area	Category	Gender	Age	Date of symptoms or confirmation	Contacts in last 24 h	Tested	Found positive	Developed symptoms on day 14	Transmission percentage
1	Palandari	Symptomatic	Male	50	May 30, 2020	42	25	0	0	0
2	Rawalakot	Symptomatic	Male	21	Aug 01, 2020	32	21	0	0	0
3	Rawalakot	Symptomatic	Male	66	Jul 08, 2020	56	28	1	1	1.8
4	Hajira	Symptomatic	Female	47	Jun 16, 2020	27	11	3	3	11.1
5	Rawalakot	Asymptomatic	Male	29	May 22, 2020	54	13	0	0	0
6	Rawalakot	Asymptomatic	Female	27	Jun 02, 2020	37	13	0	0	0
7	Rawalakot	Asymptomatic	Male	50	Sep 12, 2020	26	8	0	0	0
8	Rawalakot	Asymptomatic	Male	39	Jul 14, 2020	23	9	1	0	4.3

*Case 1: Symptomatic, transmission not traced* A 45-year-old male working as a mill laborer returned to work from his hometown after Eid-ul-Fitr holidays on May 29. This patient made contact with 42 coworkers upon returning to his employment, living in a shared mess area and lavatory. After about 27 h, the patient developed symptoms. A roommate of him suspected COVID-19 infection and informed hospital officials. The patient was admitted to the hospital where he was tested positive and stayed for 19 days before complete recovery. Meanwhile, all 42 contact persons were quarantined for 14 days. 25 of the coworkers were tested at random on day 7. All the 25 were found negative and none of the entire 42 showed any symptoms after day 14. Even after 150 days, none of the persons was found infected (transmission rate of 0%, see Table 3).

*Case 2: Symptomatic, transmission not traced* A 21-year-old male student from a village near Rawalakot became symptomatic the morning of August 1, 2020, just a day after Eid-ul-Adha. It is believed that he became infected while in Rawalpindi. In the 24 h prior to developing symptoms, he contacted 32 persons without any precautionary measures including family members and relatives. After the appearance of fever, he quarantined himself. He was brought to the hospital the next day where he was found positive for COVID-19 on PCR test and was hospitalized. All the 32 individuals who had close contact with him were kept in isolation immediately and 21 of them were selected randomly for testing after 8 days. All tested persons (including the patient’s family members) were found negative. None of the 32 showed any symptom after 14 days and 70 days (the latter being the date of data collection). The patient recovered and was discharged from the hospital after 12 days. Transmission rate was 0% in this case (Table 3).

*Case 3: Symptomatic, transmission traced* A 66-year-old male showed symptoms on July 8, 2020 and was found positive on PCR test. Just before the appearance of symptoms, on the same day, he visited 56 relatives and family members residing in Rawalakot and had intimate contact history. At about 9 pm, he felt fever and chest pain which became severe. The next morning he was brought to the hospital where he was tested positive for COVID-19 and was subsequently admitted. After 22 days of hospital stay, he recovered and was discharged from the hospital. All 56 contacted persons were self-quarantined at home and 28 of them went for a PCR test. Only 1 contacted individual developed symptoms after 9 days (also testing positive) while all 27 others tested negative. The transmission frequency was calculated to be 1.8% in this case (Table 3).

*Case 4: Symptomatic, transmission traced* A female patient of 47-years in Hajira, district Poonch AJK. Infection was likely communicated during a hospital stay. The patient was at home on June 15 and 16, where she met many family members before and during the appearance of symptoms on June 16. Of the contacted persons, 27 relatives were successfully traced. The patient tested positive and recovered after 16 days of hospitalization. All 27 contacted persons isolated themselves and 11 close relatives were tested for COVID-19. Among the close relatives remaining with the patient even after symptoms had appeared, the patient’s husband and 2 daughters tested positive. However, neither daughter developed symptoms while the husband became symptomatic 2 days after the patient. The remaining 24 persons did not develop any symptom after 14 days and even after 84 days of contact. The rate of transmission in this case was calculated to be 11.1% (Table 3).

*Case 5: Asymptomatic, transmission not traced* A 29-year-old male patient was found positive when tested after contacting a COVID-19 patient and was immediately quarantined for 14 days. 54 contacts were traced on the day he was tested, all of whom later self-isolated. After 14 days, neither the positive-tested patient, nor any of the 54 contacted persons had any symptom. For more confirmation, 13 of the contacted persons were randomly tested but all of them were found negative in PCR test. The patient tested negative and was released from quarantine after 15 days. This case was designated as an asymptomatic patient and no infection transmission was traced from him even after 140 days (Table 3).

*Case 6: Asymptomatic, transmission not traced* A 27-year-old female was tested because she was living with her husband who was symptomatic and was tested positive. The woman did not have any symptom but was found positive for COVID-19. She and 37 relatives who have met her recently were quarantined at their homes for 14 days where they remained asymptomatic. Moreover, 13 additional persons who were in close contact with the patient were tested after a week but were all negative. All 37 contacted persons along with the patient never developed any of the COVID-19 symptoms even after 130 days. The transmission frequency in this case was 0% (Table 3).

*Case 7: Asymptomatic, transmission not traced* A 50 year old school teacher was found positive during random testing on September 12, 2020. He was kept in quarantine at home immediately and 26 contacted persons were restricted to their home in isolation. After a week, 8 of family members were tested. All tested persons were negative and none of the 26 contacted persons showed any symptoms after day 14 and even after day 28 (date of data collection). The transmission frequency in this case was found to be 0% (Table 3).

*Case 8: Asymptomatic, transmission traced* This was the only asymptomatic case through which a probable transmission was traced. The patient was a 39-year-old businessman who tested positive on July 14, 2020. He was quarantined for 14 days, during which he demonstrated no symptoms. Before his initial test, he met 23 persons including 6 family members. All contacted persons self-quarantined in their homes after the test of initial patient. All 6 of the patient's family members and 3 others were selected for testing after 5 days. The patient’s 34-year-old brother tested positive but remained asymptomatic throughout the quarantine period. None of the 23 contacted individuals showed any symptoms even after 88 days post-contact. The transmission rate was found to be 4.3% in this case (Table 3).

## Discussion

This study intended to trace and estimate the rate of COVID-19 transmission by asymptomatic and pre-symptomatic patients in AJK, Pakistan. As many reports suggested, COVID-19 is transmissible through asymptomatic carriers [13–18, 21] and symptomatic patients before their symptoms appear [4, 19, 20]. These threatening facts pose a serious challenge to the global public health in mitigation of this pandemic [17]. The most important practice in halting disease spread is controlling transmission. As long as the virus is transmitted by asymptomatic and pre-symptomatic cases, blocking COVID-19 transmission is impossible. Moreover, there is evidence of some environmental transmission [27]. However, the proportion of asymptomatic patients and the transmission rate of asymptomatic/pre-symptomatic patients may be lower than initially thought, otherwise the disease would have infected a much larger observed percentage of populations. In the case of AJK, the majority of the local population within the current study area does not observe precautionary protocols when meeting apparently healthy individuals who may be asymptomatic carriers or pre-symptomatic patients. Following precautionary protocols (both to the letter and in spirit) is practically not expected in this region which has a huge population, high poverty ratio, overcrowded markets, overburdened public transport, and large families.

This study demonstrated very low transmission (1.12%) from pre-symptomatic patients and a single case of transmission (0.06%) from asymptomatic carriers. The current study traced all confirmed contacts of pre-symptomatic (n = 155) and asymptomatic patients (n = 46) before their infection confirmation by PCR. The approach used in this study evaluated infected patients and persons which they had contacted. In total, this involved 155 symptomatic patients, 46 asymptomatic patients and 7168 healthy contacted persons. A total of 5611 persons were traced having confirmed contact with pre-symptomatic patients, while 1557 persons were traced having contacted asymptomatic patients prior to their testing confirmation.

Only 63 out of 5611 persons contacting pre-symptomatic patients had confirmed infection (transmission rate = 1.12%). Individuals contacting pre-symptomatic patients were divided into four groups on the basis of time duration before the appearance of symptoms. It was found that out of 63 confirmed transmissions, 62 (98.4%) occurred with contacts during the period of 0–6 h before symptom appearance. About 4% of all contacts were infected in the 0–6 h period.

Only 1 out of 63 transmissions was traced in contacts made more than 6 h prior to the appearance of symptoms. Overall transmission rate in this group was 0.02%. Very rare transmission findings with this group leads to the hypothesis that pre-symptomatic patients transmit more infections as the time of symptom onset approaches. However, person-to-person contact in the closest time before the appearance of symptoms are always suspect as valid pre-symptomatic encounters. This is because undetected mild symptoms may be present in that period and the exact time of symptoms appearance may not be accurately recalled. Therefore the actual frequency of pre-symptomatic transmission may be slightly less than observed (1.12%). In order to reflect this, the closest group was defined as from 0 h instead of 1 h before the symptom onset.

Encounters with a pre-symptomatic patient 0–6 h before symptom onset had the highest transmission risk of any defined category (4%). However, contacted individuals traced in the period of 0–6 h before symptom onset are not guaranteed to catch infection- in fact, most did not. Unknown variables including preexisting risk or stronger viral exposure may explain why the 4% of individuals infected by this group were susceptible. Moreover, the majority of infected individuals were family members or close relatives who may have had close contact with patients even after appearance of initial symptoms.

Two important considerations can be drawn from the study data: (1) The actual transmission rate attributable to pre-symptomatic patients 0–6 h before symptom onset is likely less than 4%; (2) Risk of infection increases as the time of symptom onset draws nearer.

Although viral shedding does occur prior to 6 h, the transmission risk of encountering a pre-symptomatic patient who is greater than 6 h prior to symptom onset is extremely low (0.02%). Pre-symptomatic transmission has been identified in previous studies [4, 14, 15, 22], but the significance of the length of time prior to symptom onset and the transmission risk were not evaluated. This is the first attempt to have some insight into pre-symptomatic transmission frequency with relation to time before the symptoms. More studies with sophisticated analytics and robust testing will confirm the transmission dynamics through pre-symptomatic patients in relation to time.

Evaluated asymptomatic patients contacted 1557 individuals, yet only 1 was found to be infected. The transmission rate of asymptomatic patients was found to be very rare (0.06%). This singular case had close contact with one of the asymptomatic patients about 9 h before the confirmation of the patient’s infection by PCR test. However, this patient did not develop any of the symptoms and remained asymptomatic (*Case 8*). The contacted individual was the brother of the asymptomatic patient and tested positive 5 days after their contact. No probable source of infection other than his brother was traced, though other family members were unaffected.

Previous studies reported transmission of COVID-19 by asymptomatic patients [5, 6, 12, 13, 16–22]. The present study, however, identified only a singular instance of such infection. Further investigations are required to determine the actual transmission risk.

## Conclusions

The study concludes that the transmission rate of COVID-19 from pre-symptomatic patients in AJK, Pakistan is only about 1.12%. Infected individuals can start transmitting the virus about 6 h before the appearance of symptoms. Transmission risk increases as time draws nearer to symptom onset. Transmission risk remains very low in the period prior to 6 h before symptoms appearance. The transmission frequency of COVID-19 by asymptomatic carriers is extremely low (0.06%).

## Data Availability

All the data of this study is available on request from corresponding author.

## Abbreviations

COVID-19: Corona Virus Disease 2019
AJK: Azad Jammu and Kashmir
DHQ: District head quarters
SARS-CoV-2: Severe acute respiratory syndrome coronavirus 2
PCR: Polymerase chain reaction
MS: Microsoft
Min–Max: Minimum–maximum
UPR: University of Poonch Rawalakot
